# *Neobythites nanhaiensis* sp. nov. (Ophidiidae, Ophidiiformes) from the South China Sea, with morphology, mitogenome, and its phylogenetic position

**DOI:** 10.3897/zookeys.1269.175603

**Published:** 2026-02-13

**Authors:** Jia-Jie Chen, Bei-Jia Zhao, Ning-Ya Yang, Hui Zhang, You He, Han-Ye Zhang, Xiao-Dong Wang, Jun-Sheng Zhong

**Affiliations:** 1 East China Sea Fisheries Research Institute, Fisheries Science of Chinese Academy, 200090, Shanghai, China Shanghai Advanced Research Institute, Chinese Academy of Sciences Shanghai China; 2 Shanghai Universities Key Laboratory of Marine Animal Taxonomy and Evolution, Shanghai Ocean University, 201306, Shanghai, China Shanghai Universities Key Laboratory of Marine Animal Taxonomy and Evolution, Shanghai Ocean University Shanghai China; 3 Qingdao, China East China Sea Fisheries Research Institute, Fisheries Science of Chinese Academy Shanghai China; 4 Shanghai Synchrotron Radiation Facility, Shanghai Advanced Research Institute, Chinese Academy of Sciences, 201204, Shanghai, China Unaffiliated Qingdao China

**Keywords:** Mitochondrial genome, *Neobythites nanhaiensis* sp. nov., ophidiid, ocelli, otolith, phylogenetic tree, South China Sea

## Abstract

*Neobythites nanhaiensis***sp. nov**., a new cusk eel species from the South China Sea, is described based on 14 specimens collected at a depth of 400 m near Nan’an Reef. This species is readily distinguished from all congeners by the unique presence of two (rarely three) distinct black ocelli on the mid-flank, a trait absent in all other *Neobythites* species. It further lacks stripes, fin ocelli, or dark fin margins. The meristic features include dorsal-fin rays 107 (90–115), anal-fin rays 93 (80–94), pectoral-fin rays 25 (21–27), total vertebrae 62 (58–64), and 9–11 developed gill rakers. The complete mitochondrial genome (17,287 bp) was sequenced, exhibiting the typical vertebrate structure with an A+T bias of 55.1%. Phylogenetic analyses based on both the *COI* gene and the complete mitogenome robustly supported *N. nanhaiensis***sp. nov**. as a distinct monophyletic lineage, sister to *N. marginatus* in the mitogenome phylogeny. This integrative taxonomic approach, combining morphology, meristics, otolith morphology, and mitogenomic data, confirms the establishment of this new species, which lies outside any existing species group within the genus due to its unique flank ocelli.

## Introduction

The family Ophidiidae, within the order Ophidiiformes, comprises a diverse group of benthic and benthopelagic fishes commonly known as cusk-eels. These species are widely distributed across tropical and temperate marine waters, often inhabiting continental slopes and deep-sea environments. Among ophidiid genera, *Neobythites* stands out as one of the most species-rich lineages, characterized by a wide range of morphological adaptations and ecological niches.

To date, the genus *Neobythites* includes 60 valid species (Fricke et al. 2025). Early taxonomic efforts by Uiblein and Nielsen (2005) categorized 50 recognized species into three groups based on the presence and type of dorsal fin markings: those with ocelli, those with spots but no ocelli, and those without any markings. In a more recent revision, Uiblein and Nielsen (2023) further classified all 60 valid species into two major assemblages: 30 species possessing a dorsal fin ocellus, subdivided into five species groups (*australiensis*, *kenyaensis*, *longiventralis*, *steatiticus*), while several ocellated species remain ungrouped under the current scheme. The remaining 30 species lack a dorsal-fin ocellus.

Despite these efforts, the diversity and phylogenetic relationships within *Neobythites* remain incompletely resolved, particularly in under-explored regions such as the South China Sea. This region, known for its rich marine biodiversity and complex hydrological conditions, offers significant potential for discovering new species and clarifying taxonomic uncertainties. However, deep-water sampling challenges and limited genetic resources have historically constrained taxonomic progress in this area.

In recent years, the integration of morphological data with molecular tools—particularly mitochondrial genomics—has greatly enhanced the resolution of species boundaries and evolutionary relationships among teleost fishes. Complete mitogenome sequences provide valuable insights into genomic architecture, nucleotide composition, codon usage, and evolutionary pressures, all of which can support species delineation and phylogenetic placement.

In this study, we describe a new species of *Neobythites* collected from the South China Sea near Nan’an Reef at a depth of 400 m. A detailed morphological description is provided, supported by meristic, osteological, and otolith characteristics, along with the assembly and annotation of its complete mitochondrial genome. Furthermore, phylogenetic trees were reconstructed based on both the *COI* gene and the full mitogenome to elucidate the species’ systematic position within Ophidiidae. This integrative approach not only validates the recognition of *Neobythites nanhaiensis* sp. nov. but also contributes to a deeper understanding of the morphological and molecular evolution of deep-sea cusk-eels.

## Materials and methods

### Sample collection and DNA extraction

Fourteen specimens of an unidentified species (voucher nos. ECSFRI 28757–28760, 28765, 28843–28851) were collected by bottom trawling (cod-end mesh size: 4 cm) in the South China Sea near Nan’an Reef (5°23.08'N, 112°11.41'E) at a depth of 400 m in March 2025. For molecular analysis, muscle tissue (~ 10 × 10 mm^2^) was excised from below the right dorsal fin of fresh specimens prior to fixation. The specimen surface was cleaned with 100% ethanol before tissue collection. Total genomic DNA was extracted from the collected tissue using the TIANamp Genomic DNA Kit (TIANGEN, Beijing, China). Following tissue sampling, all specimens were fixed in 10% formaldehyde and subsequently transferred to 70% ethanol for long-term preservation.

The specimens were obtained as trawl bycatch and were deceased upon retrieval. No live animals were collected or sacrificed specifically for this study. As the research involved deceased specimens acquired through lawful fishing activities, it fulfils the criteria for exemption from specific ethical approval requirements under applicable national guidelines administered by the Ministry of Agriculture and Rural Affairs of China.

### Morphological study

Specimen photographs were taken with a digital single-lens reflex (DSLR) camera (Canon EOS 5D Mark IV) equipped with a macro lens (Canon EF 100 mm f/2.8L). Images were captured under consistent, diffuse lighting conditions against a neutral background, with a scale bar included in each frame for reference.

Morphometric measurements were taken using dissection microscopes (Leica M80 and Nikon SMZ745T) and a 300 mm digital caliper and recorded to the nearest 0.01 mm. Standard length (SL) was measured from the anteriormost tip of the snout (premaxilla) to the posterior margin of the hypural plate; head length (HL) was measured from the snout tip to the posterior-most margin of the opercle. These measurements (SL, HL) are used throughout the text unless otherwise stated. Vertebral counts were obtained from X-radiographs generated using a lab-assembled radiography system operated at 40 kV, with an effective pixel size of 9 μm. These counts further verified from cleared and stained specimens.

The employed terminology and methodology follow Nielsen (2002) and Ohashi et al. (2012), with vertebral counts determined according to Nielsen et al. (1999) and otolith description and measurement following Lin and Chang (2012) and Uiblein and Nielsen (2023). All examined specimens are deposited at the East China Sea Fisheries Research Institute (**ECSFRI**) in Shanghai, China.

### Clearing and staining

One specimen (ECSFRI 28849) was cleared and stained for bone (red) and cartilage (blue) following a protocol adapted from Dingerkus and Uhler (1977), Taylor and Van Dyke (1985) and Saruwatari et al. (1997). Briefly, the specimen was fixed in 10% formaldehyde, then transferred to an enzymatic clearing solution containing trypsin. Staining was performed sequentially with Alcian Blue 8GX for cartilage and Alizarin Red S for bone, followed by differential clearing in graded glycerin solutions to render soft tissues transparent while retaining skeletal stains.

### Mitogenome sequencing and assembly

DNA libraries were prepared with the Illumina TruSeqTM DNA Sample Preparation Kit (Illumina, USA) following manufacturer guidelines. Novogene Bioinformatics Technology Co., Ltd. (China) conducted sequencing on a DNBSEQ-T7 platform, generating 150 bp paired-end reads yielding ~5 Gb raw data per sample. Data preprocessing involved Fastp v. 0.23.2 (Chen et al. 2018) with default parameters and quality assessment using FastQC v. 0.12.1 (http://www.bioinformatics.babraham.ac.uk/projects/fastqc/). Mitogenome assembly employed the FastMitoAssembler pipeline, augmented by GetOrganelle v1.7.7 (Jin et al. 2020) and NovoPlasty v. 4.3.5 (Dierckxsens et al. 2017).

### Mitogenome annotation and sequence analyses

The complete mitochondrial genome was annotated using MitoFish (Iwasaki et al. 2013). PhyloSuite v. 1.2.3 (Zhang et al. 2020) was employed to quantify base composition, codon usage, and relative synonymous codon usage (RSCU) of protein-coding genes (PCGs). Nucleotide skewness (A+T skew = [A%−T%]/[A%+T%]; G+C skew = [G%−C%]/[G%+C%]) was calculated following Perna and Kocher (1995). Selective pressure assessed by calculating the ratio of nonsynonymous to synonymous substitutions (*Ka*/*Ks*) for protein-coding genes (PCGs) using DnaSP 6.0 (Rozas et al. 2017). The resulting *Ka*/*Ks* values were then visualized using Origin 8. Transfer RNA secondary structures were predicted using MITOS2 (Arab et al. 2017; Donath et al. 2019) and visualized using Python-based bioinformatics packages.

### Phylogenetic analysis

Phylogenetic analysis was conducted based on the *COI* gene using five sequences from the complete mitogenome generated in this study together with 53 ophidiid sequences retrieved from NCBI (Suppl. material 3: table SS1). The resulting *COI* was inferred under the best-fit substitution models GTR+F+I+G4 for Bayesian inference (BI) and K3Pu+F+I+G4 for maximum likelihood (ML). The *COI* gene sequences were aligned using MAFFT v. 7.313 (Katoh and Standley 2013), trimmed to remove ambiguous sites, resulting in a final alignment length of 511 base pairs for subsequent phylogenetic and genetic distance analysis.

A phylogeny of Ophidiidae was reconstructed using a dataset of 27 complete mitogenomes, comprising 23 species, with *Barathronus diaphanous* and *Cataetyx rubrirostris* designated as outgroups. Mitogenomic sequences obtained from GenBank (Suppl. material 3: table SS1) were processed using PhyloSuite v. 1.2.3 (Zhang et al. 2020). For phylogenetic reconstruction, all 37 mitochondrial genes (13 PCGs, 2 rRNAs, and 22 tRNAs) from each mitogenome were extracted and concatenated. The PCGs were aligned codon-by-codon using MAFFT v. 7.313 (Katoh and Standley 2013), while the rRNA and tRNA genes were aligned using the same software with default parameters. Best partitioning scheme and evolutionary models for 37 pre-defined partitions were selected using PartitionFinder2 v. 2.1.1 (Lanfear et al. 2017), with greedy algorithm and AICc criterion (Suppl. material 3: table SS2).

Maximum likelihood analysis was performed with IQ-TREE v. 2.2.0 under edge-linked partition models and supported with 5,000 ultrafast bootstrap replicates. Bayesian inference was conducted using MrBayes v. 3.2.7a (Ronquist et al. 2012), running two independent analyses for 2 × 10^6^ generations each. Resulting trees were visualized and annotated in iTOL v. 6 (Letunic and Bork 2016).

We sequenced the mitogenomes of five specimens (Suppl. material 3: table SS2) and found their structures to be identical. We therefore present the sequence from specimen ECSFRI 28760 (Accession no. PX512822) as a representative for the species.

### Genetic distance analysis

Pairwise genetic distances for the *COI* gene (barcoding region) were calculated using the p-distance model in MEGA 11 (Tamura et al. 2021) based on a subset of the trimmed alignment containing only species of the genus *Neobythites*. The resulting full distance matrix is provided as Suppl. material 2, and a summary of distances between *N. nanhaiensis* sp. nov. and all congeners is presented in Table 1. The intraspecific distance within the five sequenced specimens of *N. nanhaiensis* ranged from 0.0% to 1.0%, confirming their conspecificity. However, *N. nanhaiensis* exhibited substantial genetic divergence from all congeneric species, with p-distances ranging from 13.7–21.3 (mean ± SD = 17.0 ± 1.7%). These values far exceed the typical thresholds for interspecific delimitation in teleost fishes using *COI*, providing robust molecular support for the recognition of *N. nanhaiensis* as a distinct species.

**Table 1.** Pairwise *COI* gene (p-distance) genetic distances for *Neobythites nanhaiensis* sp. nov. and congeners.

Species / Group	Distance Range (%)	Mean ± SD (%)	*n* (pairwise)
*N. nanhaiensis* sp. nov. (intraspecific)	0.0–1.0	0.5 ± 0.4	10
*N. analis* Barnard, 1927	17.6–18.0	17.8 ± 0.2	25
*N. bimaculatus* Nielsen, 1997	14.7–15.1	14.9 ± 0.2	5
*N. gilli* Goode & Bean, 1885	15.5–15.9	15.7 ± 0.2	5
*N. longipes* Smith & Radcliffe, 1913	17.2–17.4	17.3 ± 0.1	5
*N. marginatus* Goode & Bean, 1886	16.6–17.0	16.8 ± 0.2	10
*N. sivicol*a (Jordan & Snyder, 1901)	19.2–20.3	19.8 ± 0.4	40
*N. soelae* Nielsen, 2002	13.7–14.1	13.9 ± 0.2	5
*N. steatiticus* Alcock, 1894	18.0–18.2	18.1 ± 0.1	10
*N. stelliferoides* Gilbert, 1890	15.1–15.8	15.5 ± 0.3	30
*N. stigmosus* Machida, 1984	19.2–20.3	19.6 ± 0.5	35
*N. unimaculatus* Smith & Radcliffe, 1913	18.2–21.3	19.6 ± 1.2	20
All Congeners (min–max)	13.7–21.3	17.0 ± 1.7	–

## Taxonomic account

### Neobythites nanhaiensis sp. nov.

Taxon classificationAnimaliaOphidiiformesOphidiidae

2A1B3826-5722-5186-B853-750775B00D55

https://zoobank.org/C801EF1D-0B53-4948-8215-041B2FF36D5A

Figs 1, 2

####  

English name: South China Sea cusk

Chinese name: 南海新鼬鳚

#### Type material.

**Figure 1.**
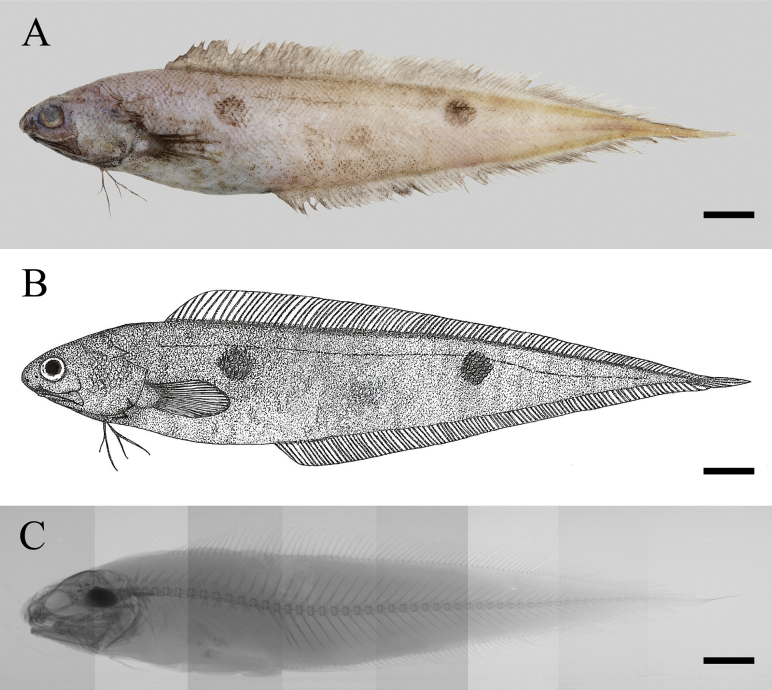
Holotype of *Neobythites nanhaiensis* sp. nov., ECSFRI 28760, 135.70 mm SL. **A**. Fresh coloration; **B**. Illustration; **C**. X-ray photograph (composite image). Scale bars: 10 mm.

**Figure 2.**
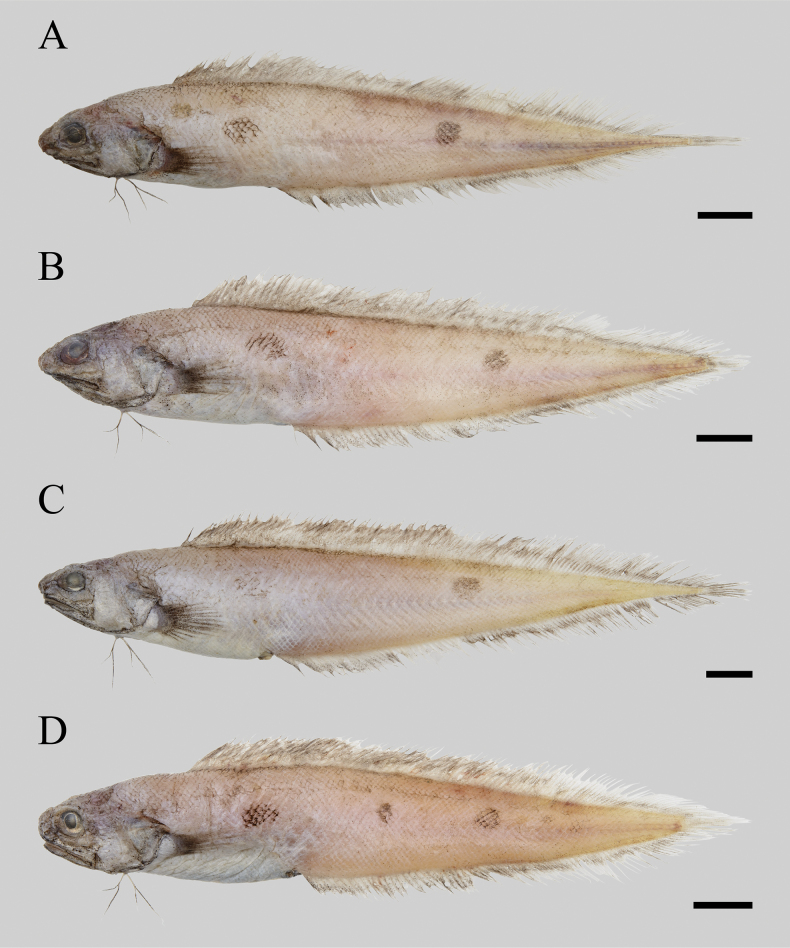
Fresh coloration of paratypes of *Neobythites nanhaiensis* sp. nov. (all in left lateral view unless specified). **A**. ECSFRI 28757, 119.52 mm SL; **B**. ECSFRI 28758, 121.26 mm SL; **C**. ECSFRI 28759, 144.51 mm SL; **D**. ECSFRI 28765, 106.32 mm SL (after thawing, mirror-reversed). Scale bars: 10 mm.

***Holotype*** • ECSFRI 28760, 135.70 mm SL, sex uncertain, South China Sea, near Nan’an Reef (5°23.08'N, 112°11.41'E), depth 400 m, collected by trawling by Bei-Jia Zhao in March 2025 (Fig. 1). ***Paratypes*** • ECSFRI 28757, 119.52 mm SL; ECSFRI 28758, 121.26 mm SL; ECSFRI 28759, 144.51 mm SL; ECSFRI 28765, 106.32 mm SL; ECSFRI 28843, 133.65 mm SL, female; ECSFRI 28844, 142.56 mm SL, male; ECSFRI 28845, 132.23 mm SL, female; ECSFRI 28846, 117.43 mm SL, female; ECSFRI 28847, 126.42 mm SL, female; ECSFRI 28848, 122.42 mm SL, female; ECSFRI 28849, 114.73 mm SL, female (cleared and stained); ECSFRI 28850, 118.44 mm SL; ECSFRI 28851, 106.29 mm SL, female. All collected with holotype. Sex uncertain except where noted (Fig. 2).

#### Diagnosis.

*Neobythites nanhaiensis* is distinguished from all currently known congeners by the presence of two (rarely three) distinct black ocelli on the mid-flank (vs flank ocelli absent in all other *Neobythites* species) (Table 2). It further differs from other ocellated species (which possess ocelli on the dorsal fin) by the absence of ocelli, stripes, or dark margins on all median and paired fins. Additionally, it can be identified by the following combination of meristic features: dorsal-fin rays 107 (90–115), originating above 4^th^ vertebra; anal-fin rays 93 (80–94), originating below 16^th^ dorsal-fin ray (13^th^–16^th^) and 20^th^ vertebra (17^th^–21^st^); pectoral-fin rays 25 (21–27); pelvic-fin rays 2; caudal-fin rays 8 (6–10); total vertebrae 62 (58–64), precaudal vertebrae 13, caudal vertebrae 49 (not including ural centra); long gill rakers on outer face of first arch 9–11; pseudobranchial filaments 4–8; preopercular spines 2; snout shorter than horizontal eye window; pelvic-fin length 7.49–11.57% SL; longest gill filament 5.27–7.53% HL; vomerine tooth patch triangular with each side concave; body yellowish to light brownish-yellow, with two (rarely three) black ocelli on flank.

**Table 2.** Morphological and meristic comparison of *Neobythites nanhaiensis* sp. nov. with selected congeners.

Character	*Neobythites nanhaiensis* sp. nov.	*N. marginatus*	*N. steatiticus*	*N. unimaculatus*
Flank ocelli	2 (rarely 3), distinct	Absent	Absent	Absent
Dorsal-fin ocellus	Absent	Absent	Present	Present
Body stripes/bars	Absent	Absent	Absent	Absent
Dorsal-fin rays	90–115	103–116	88–94	90–97
Anal-fin rays	80–94	89–97	73–77	73–77
Pectoral-fin rays	21–27	24–28	24–27	24–27
Developed gill rakers	9–11	11–14	11–14	7–9
Total vertebrae	58–64	61–66	53–57	51–52
Known depth range (m)	ca. 400	75–935	195–460	110–576
Type locality	South China Sea	Western Atlantic	Indian Ocean	Eastern Indian Ocean, western Pacific

*Neobythites nanhaiensis* sp. nov. is distinguished from other regional congeners by a suite of meristic and morphological characters. Most notably, it possesses two (rarely three) distinct ocelli on the mid-body flank, a feature absent in *N. marginatus*, *N. steatiticus*, and *N. unimaculatus*. Furthermore, the new species lacks a dorsal-fin ocellus, which is present in both *N. steatiticus* and *N. unimaculatus*. In terms of fin-ray counts, *N. nanhaiensis* has 90–115 dorsal-fin rays and 80–94 anal-fin rays, overlapping with but extending to higher counts than its congeners (Table 2). The count of developed gill rakers (9–11) is lower than in *N. marginatus* and *N. steatiticus* (11–14) but higher than in *N. unimaculatus* (7–9). Vertebrally, the new species (58–64 total vertebrae) has a higher count than *N. steatiticus* (53–57) and *N. unimaculatus* (51–52), while overlapping with *N. marginatus* (61–66). Ecologically, *N. nanhaiensis* is currently known only from a depth of ~ 400 m in the South China Sea, distinguishing it in both depth range and geographic distribution from the compared species.

Comparative data for congeners follow Nielsen (1999), Nielsen et al. (1999, 2009), Li and Zhang (2011), Iwamoto and McCosker (2014), Uiblein and Nielsen (2018), Koeda and Ho (2019), Fricke et al. (2025); data for *N. nanhaiensis* are from the present study.

#### Description.

Morphometric measurements and counts are summarized in Table 3. Condition of holotype given first, followed by those of paratypes in parentheses, if different. Body elongate, compressed, depth at dorsal-fin origin 5.9 in SL, at anal-fin origin 6.1 in SL. Head length 5.1 (4.8–5.3) in SL; snout rounded, shorter than horizontal eye window, length 6.2 in HL; eye circular, horizontal width 4.2 (3.7–4.8) in HL; interorbital space narrow, width 6.1 in HL; postorbital length 1.7 in HL.

**Table 3.** Meristic, morphometric and otolith characters in *Neobythites nanhaiensis* sp. nov.

	Holotype	Paratypes
SL (in mm)	135.7	106.29–144.51 (123.52 ± 12.06)
**Meristic characters**		
Dorsal-fin rays	107	90–115 (100 ± 6.56)
Caudal-fin rays	8	6–10 (8 ± 1.04)
Anal-fin rays	93	80–94 (87 ± 3.95)
Pectoral-fin rays	25	21–27 (24 ± 1.92)
Precaudal vertebrae	13	13 (13 ± 0.00)
Total vertebrae	62	58–64 (61 ± 1.51)
Pseudobranchial filaments	6	4–8 (6 ± 1.21)
Dorsal-fin origin above vertebra nr.	6	5–6 (6 ± 0.44)
Anal-fin origin below dorsal fin ray nr.	20	17–21 (19 ± 1.04)
Anal-fin origin below vertebra nr.	16	13–16 (15 ± 1.01)
Developed gill rakers	—	9–11 (10.45 ± 0.82)
Total gill rakers	—	16–20 (18 ± 1.35)
Preopercular spines	2	2 (2 ± 0.00)
**Morphometric characters in % SL**		
Head length	19.43	18.79–21.02 (19.80 ± 0.62)
Snout length	3.12	2.63–4.09 (3.49 ± 0.48)
Body depth at dorsal-fin origin	17.01	14.52–17.44 (16.05 ± 1.02)
Body depth at anal-fin origin	16.47	14.26–17.25 (16.15 ± 0.76)
Upper-jaw length	8.6	8.21–9.92 (9.12 ± 0.54)
Horizontal eye window	4.61	4.17–5.71 (4.72 ± 0.44)
Preanal distance	35.72	29.87–38.11 (35.23 ± 2.22)
Predorsal distance	20.8	19.40–25.59 (22.79 ± 1.58)
Pelvic-fin to anal-fin origin distance	25.85	19.87–27.65 (24.57 ± 2.51)
Pelvic-fin length	8.17	7.49–11.57 (9.30 ± 1.02)
Pectoral-fin length	12.77	10.24–13.38 (11.76 ± 0.94)
Pelvic-fin base length	79.09	77.17–79.35 (78.31 ± 1.09)
Anal-fin base length	63.22	59.17–65.03 (62.45 ± 2.43)
Gill-filament length	—	1.03–1.47 (1.19 ± 0.14)
**Postorbital distance in % HL**		
Snout length	16.08	13.16–20.21 (17.68 ± 2.14)
Caudal length	39.83	33.06–53.68 (44.37 ± 8.70)
Upper-jaw length	44.27	41.22–49.41 (46.10 ± 2.72)
Horizontal eye window	23.75	21.00–27.16 (23.80 ± 1.69)
Gill-filament length	—	5.27–7.53 (5.97 ± 0.68)
Interorbital Length	16.43	15.14–20.43 (17.83 ± 1.32)
Postorbital distance	59.75	48.85–62.51 (58.31 ± 3.48)
**Central ocellus characters**		
Snout tip to ocellus spot distance % SL	28.3	28.89–33.54 (30.08 ± 1.38)
Vertical-scale rows covered by spot	7	5–7(6 ± 0.60)

Pectoral fin short, length 1.5 in HL, tip slightly pointed, not reaching vertical through anus. Pelvic fin inserted below hind margin of preopercle, length 2.38 in HL, tip not reaching anus; inner ray slightly longer than outer. Dorsal-fin origin above pectoral-fin, base length 1.26 (1.26–1.30) in SL; anal-fin base length 1.58 (1.54–1.69) in SL; both continuous with caudal fin. Caudal fin slender, slightly pointed, length 0.60 (0.42–0.76) in horizontal eye window. Small cycloid scales embedded on head, body, and dorsal-and anal-fin bases. Lateral line single, at upper one-fourth of body, from upper part of gill opening, indistinct posteriorly.

Mouth large, subterminal; upper-jaw length 2.3 in HL; maxilla extending beyond posterior margin of eye window; posterior end truncated. Two nostrils: anterior nostril with concave rim, situated anteroventrally to snout; posterior nostril larger, positioned anteroventrally to eye and above horizontal through anterior nostril. Symphysis of premaxillae notched and edentate (Figs 1, 2).

**Figure 3.**
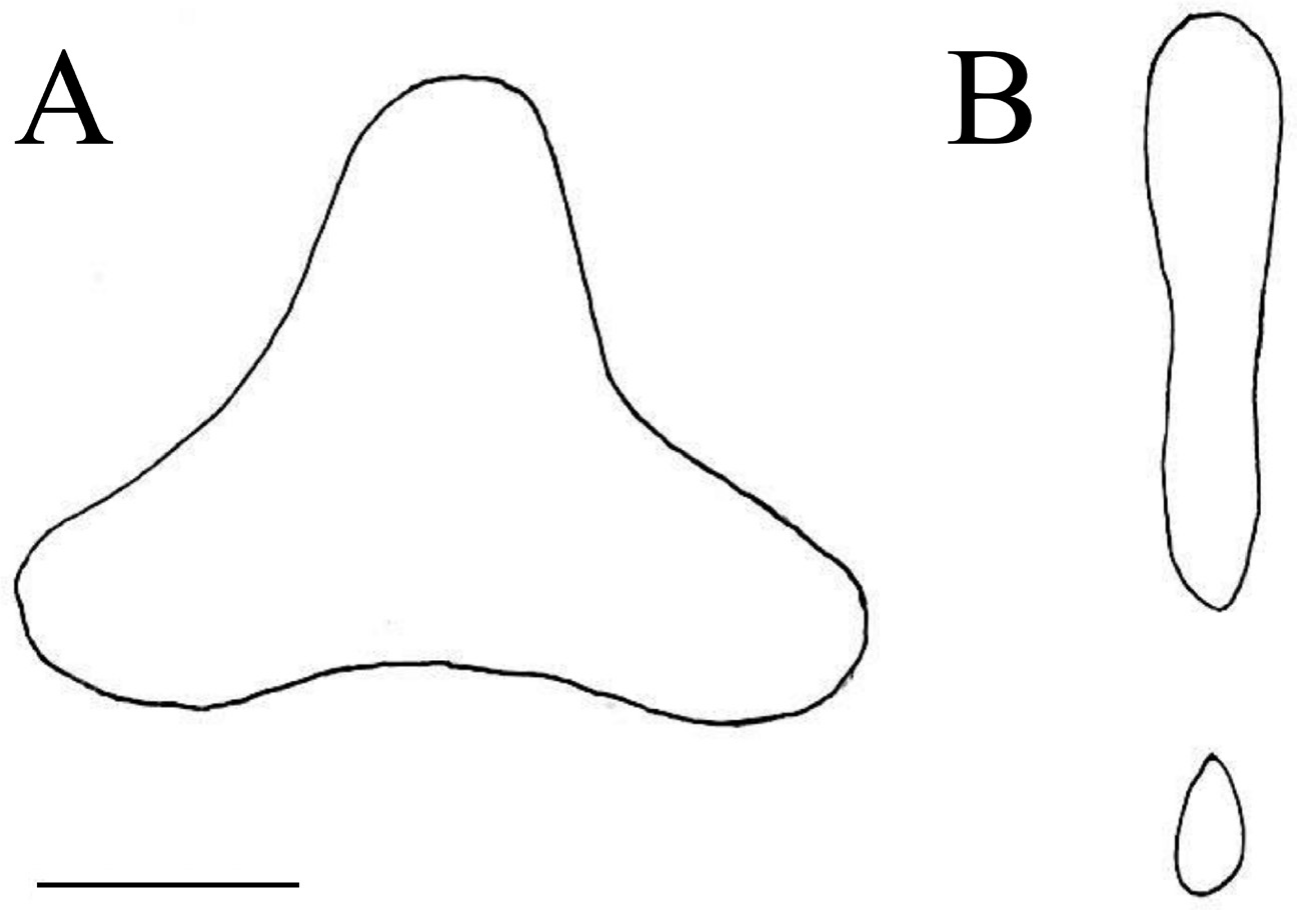
Vomerine (**A**) and basibranchial (**B**) tooth patches in *Neobythites nanhaiensis* sp. nov., paratype, ECSFRI 28845. Scale bar: 18 mm.

Premaxilla, dentary, vomer, palatine and basibranchial with villiform teeth; vomerine tooth patch triangular, each side concave (Fig. 3A); palatine tooth patch broad, gradually narrowing anteriorly; two median basibranchial tooth patches: anterior patch with broad anterior and narrow posterior part, posterior patch teardrop-shaped, 1/4 length of anterior, separated from palatine tooth patch by space equal to its length (Fig. 3B).

**Figure 4.**
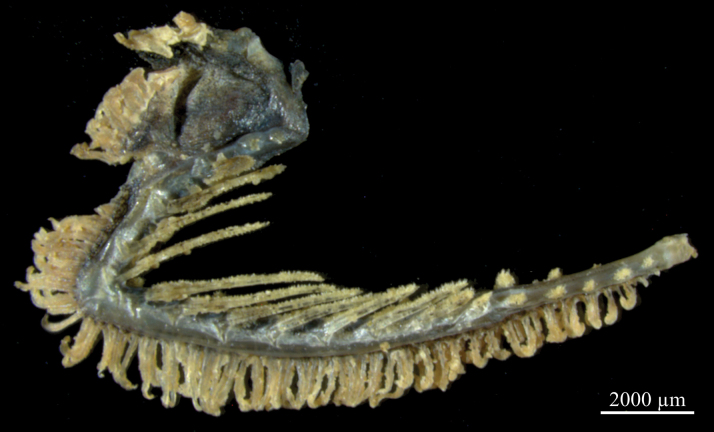
Gill rakers on first arch of *Neobythites nanhaiensis* sp. nov., paratype, ECSFRI 28759, right lateral view.

Opercle with one strong, falcated spine, its tip almost reaching posterior margin of opercle. Preopercle with two spines on posterior margin. Gill opening wide, reaching dorsal edge of pectoral-fin base. Developed gill rakers on first arch 9–11, elongated rod-shaped; pseudobranchial filaments short (Fig. 4).

**Figure 5.**
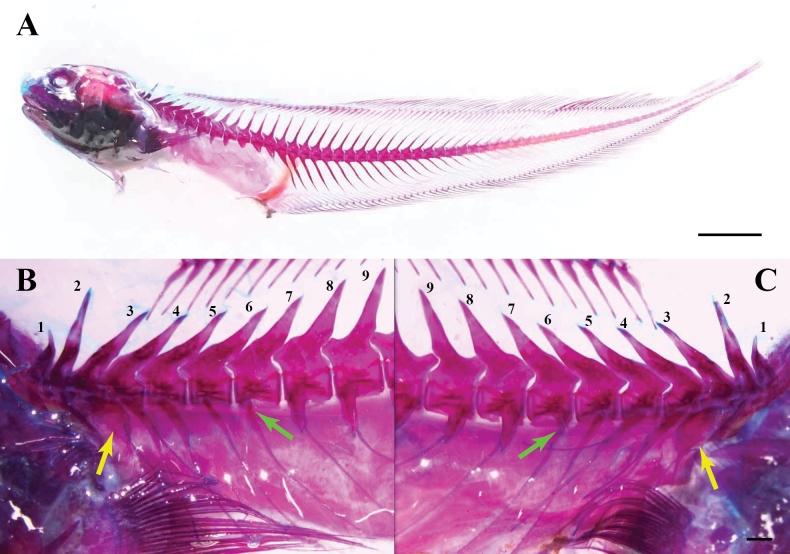
Cleared and stained specimen of *Neobythites nanhaiensis* sp. nov., paratype, ECSFRI 28849, female, 114.73 mm SL. **A**. Entire specimen, lateral view; **B, C**. Vertebrae 1–9 in left (**B**) and right (**C**) views; third vertebra showing rib with expanded base (yellow arrows) and asymmetrical parapophyses development (green arrows). Scale bars: 10 mm (**A**); 1 mm (**B, C**).

**Osteology** (Fig. 5). First neural spine short, ~1/2 length of second; bases of first three neural spines not thickened; parapophyses on vertebrae 7–13, vertebra 7 asymmetrical; pleural ribs on vertebrae 1–13: ribs 1 and 2 short rod-shaped, rib 3 with expanded base tapering to slender tip, ribs 4 and 5 rod-shaped anteriorly and slender posteriorly, ribs 6 and 7 slender; epipleurals on pleural ribs of vertebrae 3–5, that on vertebra 3 with expanded base; epineurals absent. Six anal-fin pterygiophores anterior to first haemal spine.

**Figure 6.**
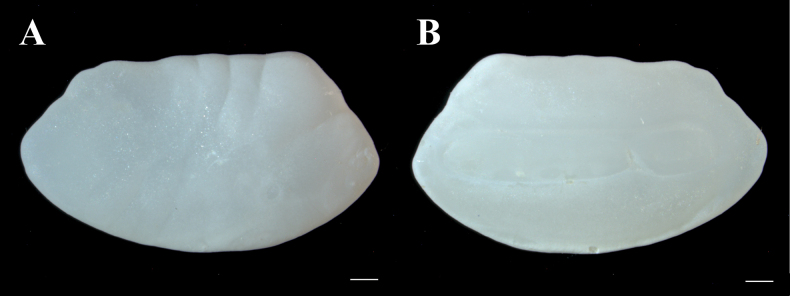
Right sagittal otolith of *Neobythites nanhaiensis* sp. nov., paratype, ECSFRI 28847, female, 8.7 mm otolith length. **A**. Distal view; **B**. Proximal view. Scale bar: 0.5 mm.

**Otolith** (Fig. 6, Table 4). Sagittal otolith oval, length 23.81–26.14% HL, length/depth ratio 1.56–1.80; ventral and anterior margins smoothly curved, dorsal margin crenate; distal surface slightly concave, proximal surface slightly convex; sulcus groove mesial, shallow, horizontal, archaesulcoid, length 16.62–20.19% HL; both colliculums present, ostial elongated, caudal oval; cristae superior and inferior poorly developed; rostrum absent; excisura absent; dorsal depression very shallow; ventral depression absent.

**Table 4.** Morphometric characteristics of the sagittal otolith.

Character	% SL	% HL	% sulcus length	% ostium length
**Otolith length**	4.78–5.44 (4.99 ± 0.20)	23.81–26.14 (25.04 ± 0.79)	—	—
**Otolith height**	2.77–3.32 (3.02 ± 0.19)	13.84–15.83 (15.14 ± 0.69)	—	—
**Sulcus length**	3.21–3.94 (3.67 ± 0.20)	16.62–20.19 (18.42 ± 1.09)	—	—
**Ostium length**	2.23–2.65 (2.49 ± 0.12)	11.14–13.62 (12.53 ± 0.70)	61.34–81.95 (68.22 ± 5.37)	—
**Ostium height**	0.70–0.90 (0.78 ± 0.06)	3.50–4.54 (3.90 ± 0.28)	18.74–24.63 (21.23 ± 1.85)	27.95–37.40 (31.21 ± 2.86)

#### Coloration.

When fresh (Figs 1A, 2), body yellowish to pale brownish-yellow, with two black ocelli on flank (rarely three, ECSFRI 28765, Fig. 2D): first ocellus between pectoral-fin tip and above anus, slightly larger, second ocellus mid-flank below middle of dorsal fin, somewhat dorsally, third ocellus (if present) between anterior two; ocelli usually covering 5–7 vertical scale rows; dorsal and anal fins dusky with pale bases, without ocellus; pectoral, pelvic and caudal fins dusky. When preserved, color similar but paler. Oral cavity pale or with sparse melanophores, including underside of tongue, lower gill arches and rakers; mouth roof behind vomer, inner opercle, and upper gill arches dusky; peritoneum black.

#### Distribution.

Known only from the type locality in the South China Sea near Nan’an Reef (5°23.08'N, 112°11.41'E), at a depth of 400 m.

#### Etymology.

The specific name *nanhai* is named after the South China Sea, known as ‘Nan Hai’ (南海) in Chinese, which is the currently known only locality for this species, from which the type specimens were collected near Nan’an Reef.

#### Common name.

Both the Chinese and English common names of this species are derived from the South China Sea, its only known habitat. Thereby, the names “南海新鼬鳚” (Nán Hăi Xīn Yòu Wèi) and “South China Sea cusk” are established, consistent with the geographical origin referenced in the specific name *nanhai*.

##### Mitogenomic characteristics

**Figure 7.**
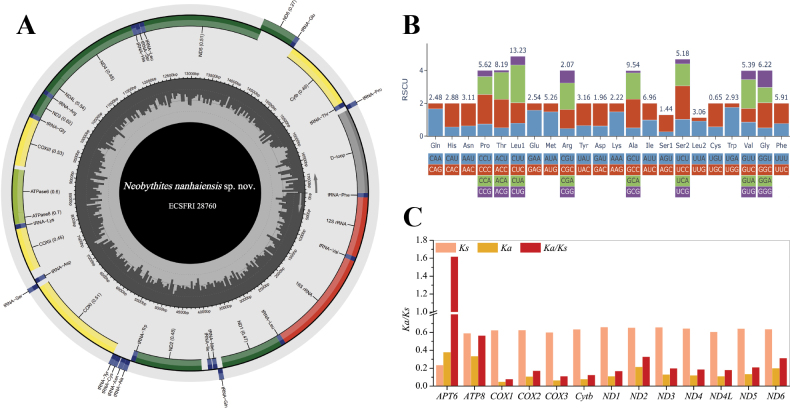
Mitogenomic characteristics of *Neobythites nanhaiensis* sp. nov. (PX512822, voucher no. ECSFRI 28760). **A**. Mitogenome of *Neobythites nanhaiensis* sp. nov.. Gene colors: ND (light blue), *COX* (orange), *ATP* (pink), *tRNA* (dark blue), *rRNA* (green), D-loop (gray). Parentheses show asymmetry indices. Gene order typical of vertebrates; **B**. Relative synonymous codon usage (RSCU) across all protein-coding genes; **C**. *Ka*, *Ks*, and *Ka*/*Ks* estimated for each PCG using sequences from 25 Neobythitinae mitogenomes.

The complete mitochondrial genome of *Neobythites nanhaiensis* sp. nov. (accession no. PX512822, ECSFRI 28760) is 17,287 bp in length and exhibits the typical vertebrate structure, comprising 13 protein-coding genes (PCGs), 22 transfer RNA (tRNA) genes, two ribosomal RNA (rRNA) genes, and one control region. All genes are encoded on the heavy strand except for *ND6* and eight tRNAs. The overall base composition shows a distinct A+T bias (55.1%). Most PCGs initiate with the standard ATG codon, except *COX1* (GTG), *ND1*, and *ND3* (ATA). A subset of PCGs possesses incomplete stop codons (TA or T). Consistent with many teleosts, the mitogenome shows a codon usage bias, with leucine being the most frequently encoded amino acid. A notable structural feature is the absence of the DHU arm in tRNA-Ser, a conserved trait among metazoans (Suppl. material 1). The *Ka*/*Ks* ratio for most PCGs indicates strong purifying selection, while the notably elevated ratio for *ATP6* (1.6173) suggests potential positive selection or relaxed constraints (Fig. 7, Suppl. material 3: tables S4–S6).

##### Phylogenetic analysis

**Figure 8.**
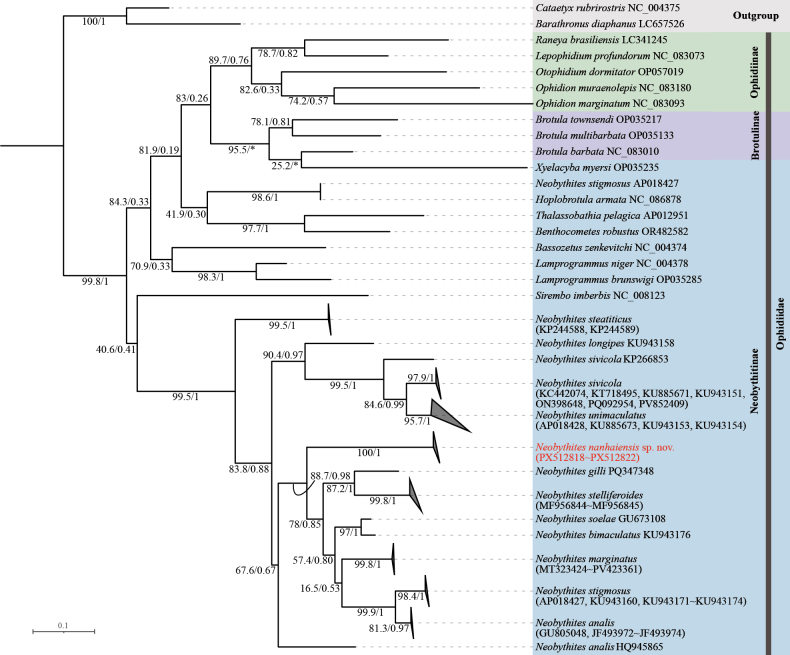
Phylogenetic tree of Ophidiidae based on *COI* sequences, analyzed by Bayesian inference (BI) and maximum likelihood (ML). Numbers above branches indicate ML bootstrap support values and Bayesian posterior probabilities, respectively; “*” indicates nodes absent from the maximum clade credibility tree. Scale bar: 0.1 substitutions per site.

Based on the *COI* gene, both maximum likelihood and Bayesian inference analyses (Fig. 8) consistently recovered *Neobythites nanhaiensis* sp. nov. as a distinct, fully supported monophyletic lineage (ML BS = 100, BI PP = 1). The species was robustly placed as the sister to a well-supported clade comprising seven congeners: *N. analis*, *N. bimaculatus*, *N. gilli*, *N. marginatus*, *N. soelae*, *N. stelliferoides*, and *N. stigmosus* (ML = 88.7, BI = 0.98). However, the internal branches within *N. nanhaiensis* showed notably variable and sometimes low support (ML BS as low as 60.7; BI PP ranging from 0.175 to 0.636), indicating some uncertainty in the fine-scale relationships among its specimens.

**Figure 9.**
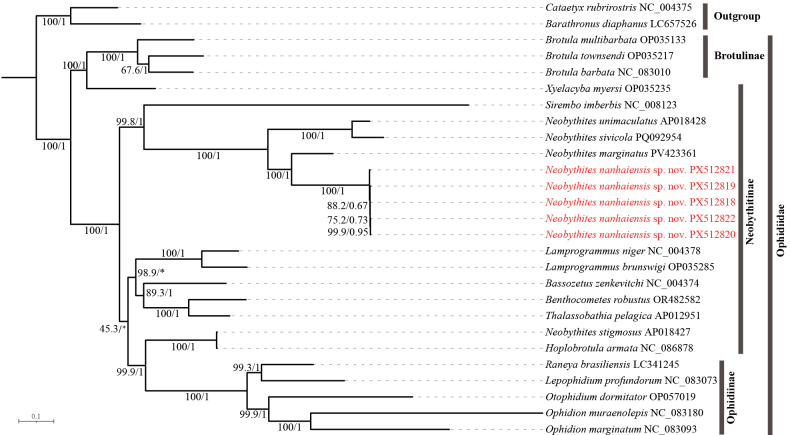
Phylogenetic tree of Ophidiidae constructed based on the amino acid sequences of the complete mitochondrial genome (concatenated set of 13 PCGs, 2 rRNAs, and 22 tRNAs) analyzed by Bayesian inference (BI) and maximum likelihood (ML). Numbers above branches indicate ML bootstrap and Bayesian posterior probabilities, respectively; “*” indicates absent from maximum clade credibility tree. Scale bar: 0.1 substitutions per site.

In contrast, phylogenetic analyses based on the concatenated set of 37 mitochondrial genes (13 PCGs, 2 rRNAs, and 22 tRNAs) (Fig. 9) provided overwhelming support for the species’ validity and internal coherence. The mitogenome (37-gene) tree robustly identified *N. nanhaiensis* as a distinct lineage and resolved it as a maximally supported sister to *N. marginatus*. Furthermore, this analysis confirmed the genetic cohesiveness of *N. nanhaiensis*, with key internal nodes receiving consistently high support (ML bootstrap values ranging from 75% to 100%; Bayesian posterior probabilities from 0.67 to 1.0).

In summary, the consistent and high support for *N. nanhaiensis* as a distinct monophyletic unit across all analyses—using both the single mitochondrial *COI* gene marker and the complete mitogenome (37 genes)— provides robust molecular evidence for its recognition as a new species. While the *COI* gene revealed some phylogenetic uncertainty at the population level, the stronger and more consistent nodal support from complete mitogenome data firmly establishes the genetic distinctness and internal coherence of *N. nanhaiensis*.

## Discussion

*Neobythites nanhaiensis* sp. nov. is readily distinguished from all other valid species of *Neobythites* by the presence of two or three distinct black ocelli on the flank. A detailed comparison with its phylogenetically proximate and morphologically similar congeners further clarifies its distinctiveness. Compared to its sister species *N. marginatus* (as inferred from mitogenome phylogeny), *N. nanhaiensis* lacks the prominent dark margins on the dorsal, anal, and caudal fins that are characteristic of *N. marginatus*, and possesses flank ocelli (vs absent). Within the clade containing *N. gilli* and *N. stelliferoides* (*COI* phylogeny), *N. nanhaiensis* differs by having distinct flank ocelli (vs body with small, irregular dark spots in *N. gilli* and numerous white spots in *N. stelliferoides*), and a higher total vertebral count (58–64, vs 53–58 in *N. gilli* and 49–52 in *N. stelliferoides*). It is distinguished from other ocellated species like *N. steatiticus*, *N. longipes*, and *N. unimaculatus* by the position of its ocelli (on the flank vs primarily on the dorsal fin) and the absence of any ocelli or dark markings on the median fins. These morphological stripes, combined with its unique ocellus pattern, firmly place it outside any species group defined by Uiblein and Nielsen (2005, 2023).

Ecologically, the new species was collected at a depth of 400 m, within the 300–450 m range where Uiblein and Nielsen (2005) observed the highest diversity of ocellus patterns in *Neobythites*. These authors also suggested that dorsal fin ocelli may aid in dynamic visual signaling, while dark margins and vertical bars could assist in predator avoidance and social communication (Barlow 1972). In contrast, *N. nanhaiensis* appears to employ neither strategy, potentially relying on whole-body display or concealment instead.

Within the genetic distance matrix, a notable discrepancy was identified concerning *Neobythites analis*. One sequence (GenBank accession number HQ945865) showed an unexpectedly high p-distance of ~15.6% when compared to the other four *N. analis* sequences (e.g., JF493972–JF493974, GU805048; pairwise distances among these four: 0.0–0.4%) (Suppl. material 2). This level of divergence far exceeds typical intraspecific variation for fish *COI* and strongly suggests that the specimen corresponding to HQ945865 represents a misidentification or a distinct, cryptic species. For the purposes of this study and based on congruence with the majority of reference sequences and current taxonomy, we consider the cluster comprising JF493972–JF493974 and GU805048 to represent the true *N. analis*. A similar anomaly was observed for one *N. stigmosus* sequence (AP018427, showing ~22% divergence from other conspecifics). These cases underscore the prevalence of undetected cryptic diversity or specimen identification errors in public databases, which can complicate phylogenetic and barcoding studies. Importantly, the high genetic distance between *N. nanhaiensis* and all congeners, including both putative forms of *N. analis*, remains substantial (≥14.0%), reinforcing the conclusion that it is a distinct species.

Molecular analyses strongly support the validity of this new species. It was consistently recovered as a highly supported monophyletic lineage across *COI* and complete mitogenome (37-gene) datasets. Notably, genome-scale analyses confirming both species-level monophyly and intraspecific cohesion (ML BS 75–100%, BI PP 0.67–1.0). In the phylogeny based on the complete mitogenome, *N. nanhaiensis* was robustly placed as sister to *N. marginatus*. It is important to note that this inference is based on a currently limited sampling of mitogenomes within the genus. This specific sister-group relationship is not recovered in the more densely sampled *COI* gene tree (Fig. 8), which instead places *N. nanhaiensis* as sister to a larger clade containing both ocellated and non-ocellated species (e.g., *N. gilli*, *N. marginatus*, *N. stelliferoides*, *N. stigmosus*). This discrepancy highlights how phylogenetic resolution, especially for deep relationships, can be sensitive to both the genetic marker used and the breadth of taxon sampling. Therefore, while the mitogenome data robustly confirms the distinctness of *N. nanhaiensis*, its precise sister-group relationship awaits clarification through future studies with expanded mitogenomic sampling.

The broader taxon sampling in the *COI* phylogeny provides a valuable complementary perspective. The finding that *N. nanhaiensis* groups with a morphologically heterogeneous assemblage underscores the complex evolutionary history of color patterns in *Neobythites* and suggests that the presence or absence of fin ocelli may not be a phylogenetically conservative trait at this genetic scale. Furthermore, the variable and sometimes low support for internal branches within the *N. nanhaiensis COI* clade may reflect very recent diversification or incipient population structure within this geographically restricted species—a signal potentially diluted in the more conserved, genome-scale mitogenome data.

Critically, both datasets congruently affirm the monophyly and distinctness of *N. nanhaiensis*. The *COI* tree, with its extensive sampling, provides a detailed view of its relationships within a broader species assemblage, whereas the mitogenome tree offers robust resolution at a deeper phylogenetic level. Together, they provide multi-layered molecular evidence supporting its status as a new species. Nevertheless, due to the still limited genetic data for the genus, the broader relationships within *Neobythites* are not fully resolved, and the current phylogenies do not entirely align with morphology-based classifications.

Beyond its phylogenetic position, the mitogenome of *N. nanhaiensis* retains the typical vertebrate structure while showing several distinctive evolutionary features, including a strong A+T bias (55.1%) consistent with teleost mitogenomes, a missing DHU arm in tRNA-Ser representing a conserved metazoan trait (Satoh et al. 2016), and codon usage biased toward leucine as the most frequent amino acid (Wolstenholme 1992). Strong purifying selection across most PCGs (*Ka*/*Ks* ≪ 1) supports functional conservation in ophidiids. In contrast, the elevated *Ka*/*Ks* in *ATP6* (highest pairwise value = 1.6173) suggests it may be under relaxed selective constraints or positive selection. While this observation is intriguing, attributing it directly to deep-sea adaptation requires caution, as *N. nanhaiensis* is a mid-slope (400 m) benthic species, not a hadal resident. The *ATP6* gene encodes a key subunit of the mitochondrial ATP synthase complex, central to cellular energy production. Instances of positive selection or accelerated evolution in mitochondrial oxidative phosphorylation (OXPHOS) genes have been documented in several deep-sea fish lineages and are often hypothesized to be metabolic adaptations to cold, hypoxic, and high-pressure environments (e.g., in hadal snailfishes; Wang et al. 2019). In this context, the elevated *Ka*/*Ks* ratio observed in the *ATP6* gene in our study may be best interpreted as a putatively adaptive signal worthy of further investigation. It could reflect lineage-specific evolutionary dynamics potentially linked to mid-slope environmental conditions, though this remains speculative. Definitive interpretation will require functional assays and population-level studies.

## Conclusions

*Neobythites nanhaiensis* sp. nov. is established as a distinct species based on a combination of morphological uniqueness, molecular phylogenetic evidence, and specific mitogenomic features. It is morphologically diagnosed by the presence of two or three flank ocelli—a trait absent in all other known *Neobythites* species—and the absence of stripes, fin ocelli, and dark fin margins, placing it outside existing species groups. Phylogenetically, it forms a well-supported monophyletic lineage. In the current mitogenome phylogeny, *N. nanhaiensis* is resolved as sister to *N. marginatus*, but this relationship should be interpreted with caution given the limited taxon sampling of mitogenomes and its inconsistency with the topology from the more densely sampled *COI* gene tree. The precise sister-group relationship of *N. nanhaiensis* within the genus awaits clarification through future studies with expanded genomic sampling. Mitogenomic analyses further reveal conserved vertebrate architecture alongside lineage-specific evolutionary signals, including strong A+T bias, tRNA-Ser structural modification, and a notably elevated *Ka*/*Ks* ratio in the *ATP6* gene. This molecular signature warrants further investigation to determine if it reflects stochastic drift, relaxed selection, or an adaptive response to the deep-sea environment. The species was collected at 400 m depth, within the zone of highest ocellus diversity reported for the genus (Uiblein and Nielsen 2005). Its unique color pattern—possessing flank ocelli while lacking dorsal-fin ocelli, stripes, and dark fin margins—differs from the morphological correlates of previously proposed visual ecological models for *Neobythites*, which involve dorsal-fin ocelli for dynamic signaling or dark margins/bars for predator avoidance and communication (Barlow 1972; Uiblein and Nielsen 2005). This integrative taxonomic approach not only validates *N. nanhaiensis* as a new species but also enhances our understanding of morphological and molecular evolution in deep-sea cusk eels.

## Supplementary Material

XML Treatment for Neobythites nanhaiensis

## Additional information

### Use of AI

No use of AI was reported.

### Author ORCIDs

Jia-Jie Chen https://orcid.org/0000-0001-7487-231X

Ning-Ya Yang https://orcid.org/0009-0002-2288-4399

You He https://orcid.org/0009-0004-8027-0760

Han-Ye Zhang https://orcid.org/0000-0002-1645-6796

Xiao-Dong Wang https://orcid.org/0000-0001-9424-7896

Jun-Sheng Zhong https://orcid.org/0000-0002-9145-7473

### Data availability

All of the data that support the findings of this study are available in the main text or Supplementary Information.

## Supplementary materials

Supplementary material 1 tRNA*Neobythites* sp. nov. Data type pdf Explanation note Secondary structure of the 22 tRNA genes of the mitochondrial genome of *Neobythites nanhaiensis* sp. nov. https://binary.pensoft.net/file/1538432This dataset is made available under the Open Database License (http://opendatacommons.org/licenses/odbl/1.0/). The Open Database License (ODbL) is a license agreement intended to allow users to freely share, modify, and use this Dataset while maintaining this same freedom for others, provided that the original source and author(s) are credited. Jia-Jie Chen, Bei-Jia Zhao, Ning-Ya Yang, Hui Zhang, You He, Han-Ye Zhang, Xiao-Dong Wang, Jun-Sheng Zhong

Supplementary material 2 Pairwise p-distance matrix Data type xlsx Explanation note Complete pairwise p-distance matrix for *COI* sequences of *Neobythites* species. https://binary.pensoft.net/file/1538433This dataset is made available under the Open Database License (http://opendatacommons.org/licenses/odbl/1.0/). The Open Database License (ODbL) is a license agreement intended to allow users to freely share, modify, and use this Dataset while maintaining this same freedom for others, provided that the original source and author(s) are credited. Jia-Jie Chen, Bei-Jia Zhao, Ning-Ya Yang, Hui Zhang, You He, Han-Ye Zhang, Xiao-Dong Wang, Jun-Sheng Zhong

Supplementary material 3 Phylogenetic data and mitochondrial genome characteristics Data type docx Explanation note **table SS1**: *COI* and Mitochondrial sequences used in the phylogenetic analysis as shown in this study. **table SS2**: Best partitioning schemes and models based on different datasets for Bayesian inference (BI) and maximum likelihood (ML) analysis. **table SS4**: Information on each gene fragment of *Neobythites nanhaiensis* sp. nov. **table S5**: Base composition of the *Neobythites nanhaiensis* sp. nov. mitochondrial genome. **table S6**: Codon number and RSCU of *Neobythites nanhaiensis* sp. nov. mitochondrial PCGs. https://binary.pensoft.net/file/1538434This dataset is made available under the Open Database License (http://opendatacommons.org/licenses/odbl/1.0/). The Open Database License (ODbL) is a license agreement intended to allow users to freely share, modify, and use this Dataset while maintaining this same freedom for others, provided that the original source and author(s) are credited. Jia-Jie Chen, Bei-Jia Zhao, Ning-Ya Yang, Hui Zhang, You He, Han-Ye Zhang, Xiao-Dong Wang, Jun-Sheng Zhong
