# Supplementary material for: Neobythites nanhaiensis sp. nov. (Ophidiidae, Ophidiiformes) from the South China Sea, with morphology, mitogenome, and its phylogenetic position
Source: Zookeys. 2026 Feb 13;1269:107–28. doi: 10.3897/zookeys.1269.175603 (PMC12924049; doi:10.3897/zookeys.1269.175603)
Supplement: Supplementary material 1 — tRNANeobythites sp. nov. [file zookeys-1269-107_article-175603__-s001.pdf]

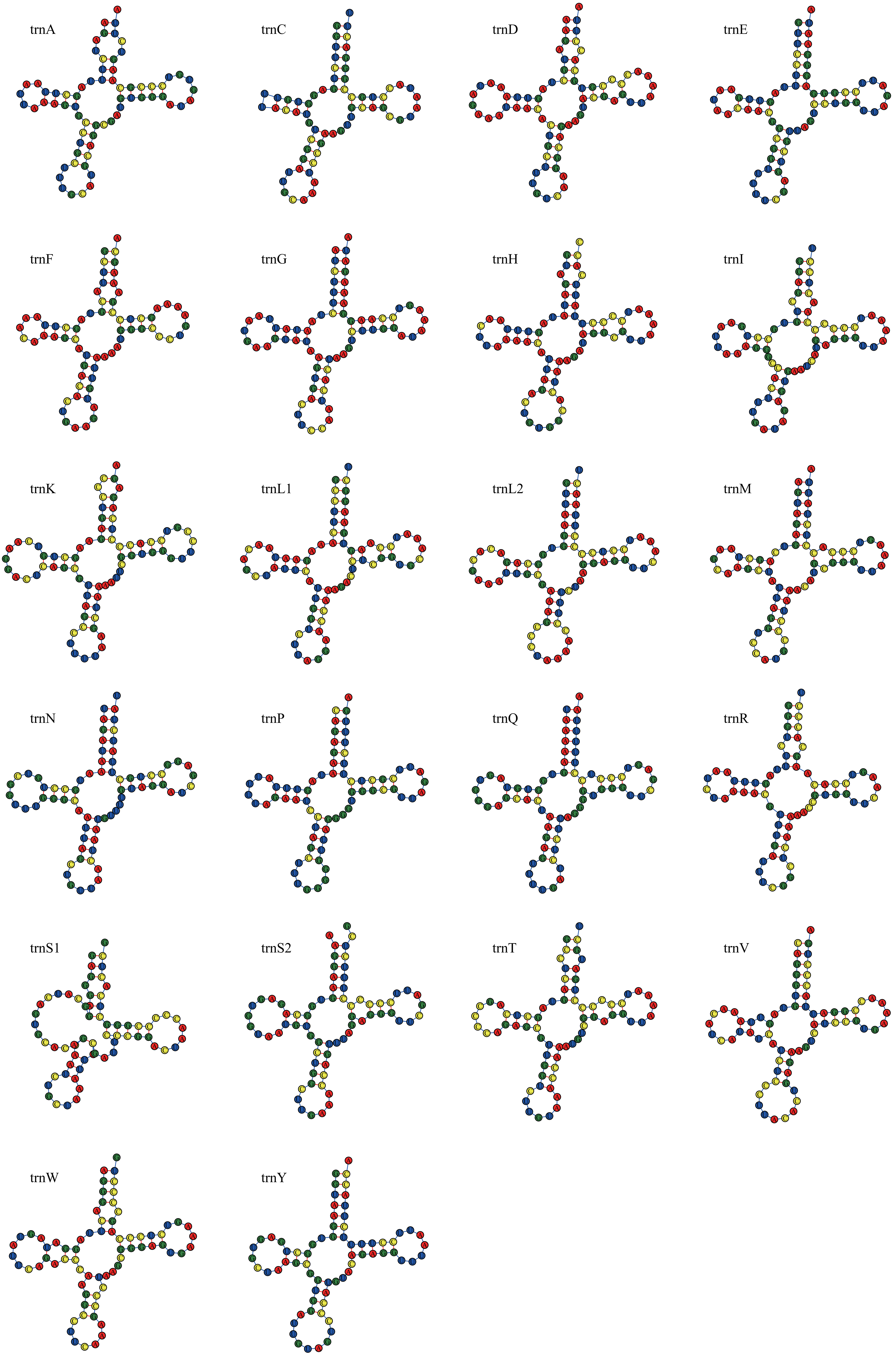
trnA
trnC
trnD
trnE
trnF
trnG
trnH
trnI
trnK
trnL1
trnL2
trnM
trnN
trnP
trnQ
trnR
trnS1
trnS2
trnT
trnV
trnW
trnY
