# Supplementary material for: Neobythites nanhaiensis sp. nov. (Ophidiidae, Ophidiiformes) from the South China Sea, with morphology, mitogenome, and its phylogenetic position
Source: Zookeys. 2026 Feb 13;1269:107–28. doi: 10.3897/zookeys.1269.175603 (PMC12924049; doi:10.3897/zookeys.1269.175603)
Supplement: Supplementary material 3 — Phylogenetic data and mitochondrial genome characteristics [file zookeys-1269-107_article-175603__-s003.docx]

**Table S1.** List of *COI* and mitogenome sequences used in this study. Sequences marked with an asterisk (*) were included in the genus-level genetic distance calculation.

| **Species** | ***COI*** | **Mitogenome** | **Voucher** | **Locaion** | **Reference** |
| --- | --- | --- | --- | --- | --- |
| **Out group** |  |  |  |  |  |
| *Barathronus diaphanus* | LC657526 | LC657526 | NSMT:P:111903 |  | Song et al. 2025 |
| *Cataetyx rubrirostris* | NC_004375 | NC_004375 |  |  | Miya et al. 2003, Song et al. 2025 |
| **Ophidiidae** |  |  |  |  |  |
| **Brotulinae** |  |  |  |  |  |
| *Brotula barbata* | NC_083010 | NC_083010 | USNM:FISH:400732 | Costa Rica, Atlantic Ocean | Song et al. 2025 |
| *Brotula multibarbata* | OP035133 | OP035133 | USNM:FISH:446410 | Wallis and Futuna, Futuna | Song et al. 2025 |
| *Brotula townsendi* | OP035217 | OP035217 | USNM:FISH:446238 | Wallis and Futuna, Alofi | Song et al. 2025 |
| **Neobythitinae** |  |  |  |  |  |
| *Bassozetus zenkevitchi* | NC_004374 | NC_004374 |  |  | Miya et al. 2003, Song et al. 2025 |
| *Benthocometes robustus* | OR482582 | OR482582 | USNM:FISH:455343 | Atlantic Ocean, USA | Song et al. 2025 |
| *Hoplobrotula armata* | NC_086878 | NC_086878 |  |  | Song et al. 2025 |
| *Lamprogrammus brunswigi* | OP035285 | OP035285 | USNM:FISH:407327 | Atlantic Ocean, Panama | Song et al. 2025 |
| *Lamprogrammus niger* | NC_004378 | NC_004378 |  |  | Miya et al. 2003, Song et al. 2025 |
| *Neobythites analis** | GU805048 |  | ADC09_96.18#4.1 | Tugela Banks, South Africa | From NCBI |
| *Neobythites analis** | HQ945865 |  | ADC10_96.18 #6 | Tugela Banks, South Africa | From NCBI |
| *Neobythites analis** | JF493972 |  | 96.22#2 | KwaZulu-Natal, Tugela Deep, South Africa | From NCBI |
| *Neobythites analis** | JF493973 |  | ACD07_96.18 #2 | South Africa | From NCBI |
| *Neobythites analis** | JF493974 |  | ACD07_96.18 #1 | South Africa | From NCBI |
| *Neobythites bimaculatus** | KU943176 |  |  | Taiwan | Chang et al. 2017 |
| *Neobythites gilli** | PQ347348 |  | FWRI20710 | USA | From NCBI |
| *Neobythites longipes** | KU943158 |  |  | Taiwan | Chang et al. 2017 |
| *Neobythites marginatus** | MT323424 |  | DPND 1348 | Gulf of Mexico, USA | From NCBI |
| *Neobythites marginatus** | PV423361 | PV423361 | USNM:FISH:407173 |  | From NCBI |
| *Neobythites nanhaiensis* sp. nov. * | PX512818 | PX512818 | ECSFRI 28757 | Nan'an Reef, South China Sea | this study |
| *Neobythites nanhaiensis* sp. nov. * | PX512819 | PX512819 | ECSFRI 28758 | Nan'an Reef, South China Sea | this study |
| *Neobythites nanhaiensis* sp. nov. * | PX512820 | PX512820 | ECSFRI 28759 | Nan'an Reef, South China Sea | this study |
| *Neobythites nanhaiensis* sp. nov. * | PX512821 | PX512821 | ECSFRI 28760 | Nan'an Reef, South China Sea | this study |
| *Neobythites nanhaiensis* sp. nov. * | PX512822 | PX512822 | ECSFRI 28765 | Nan'an Reef, South China Sea | this study |
| *Neobythites sivicola** | KC442074 |  |  |  | From NCBI |
| *Neobythites sivicola** | KP266853 |  |  |  | From NCBI |
| *Neobythites sivicola** | KT718495 |  | ASIZP0078877 | Taiwan | From NCBI |
| *Neobythites sivicola** | KU885671 |  |  | Taiwan | Chang et al. 2017 |
| *Neobythites sivicola** | KU943151 |  |  | Taiwan | Chang et al. 2017 |
| *Neobythites sivicola** | ON398648 |  | Teramura-A71 | Shizuoka, Japan | Teramura et al. 2022 |
| *Neobythites sivicola** | PQ092954 | PQ092954 |  | East China Sea | Song et al. 2025 |
| *Neobythites sivicola** | PV852409 |  | KTS_02844 | Taiwan | From NCBI |
| *Neobythites soelae** | GU673108 |  | BW-A4709 | Australia | From NCBI |
| *Neobythites steatiticus** | KP244588 |  | NBFGR:CHN:TK19 | India | From NCBI |
| *Neobythites steatiticus** | KP244589 |  | NBFGR:CHN:KN3 | India | From NCBI |
| *Neobythites stelliferoides** | MF956844 |  | USNM:FISH:421392 | Pacific Ocean | From NCBI |
| *Neobythites stelliferoides** | MF956845 |  | USNM:FISH:421399 | Pacific Ocean | From NCBI |
| *Neobythites stelliferoides** | MF956846 |  | USNM:FISH:422341 | Pacific Ocean | From NCBI |
| *Neobythites stelliferoides** | MF956847 |  | USNM:FISH:422448 | Pacific Ocean | From NCBI |
| *Neobythites stelliferoides** | MF956848 |  | USNM:FISH:422312 | Pacific Ocean | From NCBI |
| *Neobythites stelliferoides** | MF956849 |  | USNM:FISH:421439 | Pacific Ocean | From NCBI |
| *Neobythites stigmosus** | AP018427 | AP018427 | NMMBP:0200892 |  | Song et al. 2025 |
| *Neobythites stigmosus** | KU943160 |  |  | Taiwan | Chang et al. 2017 |
| *Neobythites stigmosus** | KU943171 |  |  | Taiwan | Chang et al. 2017 |
| *Neobythites stigmosus** | KU943172 |  |  | Taiwan | Chang et al. 2017 |
| *Neobythites stigmosus** | KU943173 |  |  | Taiwan | Chang et al. 2017 |
| *Neobythites stigmosus** | KU943174 |  |  | Taiwan | Chang et al. 2017 |
| *Neobythites unimaculatus** | AP018428 | AP018428 | NMMBP:020901 |  | Song et al. 2025 |
| *Neobythites unimaculatus** | KU885673 |  |  | Taiwan | Chang et al. 2017 |
| *Neobythites unimaculatus** | KU943153 |  |  | Taiwan | Chang et al. 2017 |
| *Neobythites unimaculatus** | KU943154 |  |  | Taiwan | Chang et al. 2017 |
| *Sirembo imberbis* | NC_008123 | NC_008123 |  |  | Miya et al. 2003, Song et al. 2025 |
| *Thalassobathia pelagica* | AP012951 | AP012951 |  |  | Song et al. 2025 |
| *Xyelacyba myersi* | OP035235 | OP035235 | USNM:FISH:400705 | Panama, Atlantic Ocean, | Song et al. 2025 |
| **Ophidiinae** |  |  |  |  |  |
| *Lepophidium profundorum* | NC_083073 | NC_083073 | USNM:FISH:454723 | Virginia, USA, Atlantic Ocean | Song et al. 2025 |
| *Ophidion marginatum* | NC_083093 | NC_083093 | USNM:FISH:454764 | Virginia, USA, Atlantic Ocean | Song et al. 2025 |
| *Ophidion muraenolepis* | NC_083180 | NC_083180 | USNM:FISH:409285 | Marquesas Islands, MotuIti, French Polynesia | Song et al. 2025 |
| *Otophidium dormitator* | OP057019 | OP057019 | USNM:FISH:416300 | Belize, Atlantic Ocean | Song et al. 2025 |
| *Raneya brasiliensis* | LC341245 | LC341245 |  | Province of Chubut, Rawson, Argentina | Fromm et al. 2019, Song et al. 2025 |

**References**

Chang C-H, Shao K-T, Lin H-Y, Chiu Y-C, Lee M-Y, Liu S-H, Lin P-L (2017) DNA barcodes of the native ray-finned fishes in Taiwan. Molecular Ecology Resources 17: 796–805. https://doi.org/10.1111/1755-0998.12601

Fromm A, Atkinson SD, Alama-Bermejo G, Cartwright P, Bartholomew JL, Huchon D (2019) A new mitochondrial gene order in the banded cusk-eel Raneya brasiliensis (Actinopterygii, Ophidiiformes). Mitochondrial DNA. Part B, Resources 4: 1–4. https://doi.org/10.1080/23802359.2018.1532824

Miya M, Takeshima H, Endo H, Ishiguro NB, Inoue JG, Mukai T, Satoh TP, Yamaguchi M, Kawaguchi A, Mabuchi K, Shirai SM, Nishida M (2003) Major patterns of higher teleostean phylogenies: a new perspective based on 100 complete mitochondrial DNA sequences. Molecular Phylogenetics and Evolution 26: 121–138. https://doi.org/10.1016/S1055-7903(02)00332-9

Song X, Zhang R, Zhang H, Li S, Ling J (2025) The complete mitochondrial genome of Neobythites sivicola (Jordan & Snyder, 1901) (Ophidiiformes: Ophidiidae). Mitochondrial DNA. Part B, Resources 10: 99–102. https://doi.org/10.1080/23802359.2025.2449685

Teramura A, Koeda K, Matsuo A, Sato MP, Senou H, Ho H-C, Suyama Y, Kikuchi K, Hirase S (2022) Assessing the effectiveness of DNA barcoding for exploring hidden genetic diversity in deep-sea fishes. Marine Ecology Progress Series 701: 83–98. https://doi.org/10.3354/meps14193

**Table S2** Best partitioning schemes and models based on different datasets for Bayesian inference (BI) and maximum likelihood (ML) analysis.

| **Partition names** | **Best Model** |
| --- | --- |
| P1: (ATP6_mafft) | GTR+I+G |
| P2: (ATP8_mafft) | GTR+G |
| P3: (COX1_mafft) | GTR+I+G |
| P4: (COX3_mafft, COX2_mafft) | GTR+I+G |
| P5: (Cytb_mafft) | GTR+I+G |
| P6: (ND4_mafft, ND5_mafft, ND1_mafft) | GTR+I+G |
| P7: (ND2_mafft) | GTR+I+G |
| P8: (ND3_mafft) | GTR+I+G |
| P9: (ND4L_mafft) | GTR+I+G |
| P10: (ND6_mafft) | GTR+I+G |
| P11: (12s_mafft, tRNA_Gly_mafft) | GTR+I+G |
| P12: (16s_mafft) | GTR+I+G |
| P13: (tRNA_Pro_mafft, tRNA_Asn_mafft, tRNA_Ala_mafft, tRNA_Glu_mafft) | GTR+G |
| P14: (tRNA_Phe_mafft, tRNA_Met_mafft, tRNA_Arg_mafft, tRNA_Tyr_mafft, tRNA_Cys_mafft, tRNA_Val_mafft, tRNA_Ile_mafft) | GTR+I+G |
| P15: (tRNA_Asp_mafft, tRNA_His_mafft) | GTR+G |
| P16: (tRNA_Gln_mafft, tRNA_Ser2_mafft) | GTR+I |
| P17: (tRNA_Leu2_copy2_mafft) | K80+G |
| P18: (tRNA_Leu2_mafft, tRNA_Ser2_copy2_mafft) | SYM+G |
| P19: (tRNA_Lys_mafft, tRNA_Trp_mafft, tRNA_Thr_mafft) | SYM+G |

**Table S4** Information on each gene fragment of *Neobythites nanhaiensis* sp. nov.

| **Name** | ***Neobythites nanhaiensis* sp. nov. (voucher no. ECSFRI 28760)** | | | | | |
| --- | --- | --- | --- | --- | --- | --- |
|  | **Start** | **Stop** | **Strand** | **Length** | **IGR*** | **Codons** |
| *tRNA-Phe* | 1 | 68 | H | 68 |  |  |
| *12s* | 69 | 1017 | H | 949 |  |  |
| *tRNA-Val* | 1018 | 1088 | H | 71 |  |  |
| *16s* | 1089 | 2764 | H | 1676 |  |  |
| *tRNA-Leu2* | 2765 | 2836 | H | 72 |  |  |
| *ND1* | 2837 | 3811 | H | 975 |  | ATA/TAA |
| *tRNA-Ile* | 3825 | 3895 | L | 71 | 13 |  |
| *tRNA-Gln* | 3895 | 3963 | H | 69 | -1 |  |
| *tRNA-Met* | 3974 | 4043 | H | 70 | 10 |  |
| *ND2* | 4141 | 5186 | H | 1046 | 97 | ATG/TA |
| *tRNA-Trp* | 5187 | 5258 | H | 72 |  |  |
| *tRNA-Ala* | 5260 | 5329 | L | 70 | 1 |  |
| *tRNA-Asn* | 5331 | 5403 | L | 73 | 1 |  |
| *tRNA-Cys* | 5417 | 5481 | L | 65 | 13 |  |
| *tRNA-Tyr* | 5482 | 5551 | L | 70 |  |  |
| *COX1* | 5553 | 7103 | H | 1551 | 1 | GTG/TAA |
| *tRNA-Ser2* | 7104 | 7174 | L | 71 |  |  |
| *tRNA-Asp* | 7178 | 7249 | H | 72 | 3 |  |
| *COX2* | 7387 | 8077 | H | 691 | 137 | ATG/T |
| *tRNA-Lys* | 8078 | 8151 | H | 74 |  |  |
| *atp8* | 8156 | 8332 | H | 177 | 4 | ATG/TAA |
| *atp6* | 8326 | 9018 | H | 693 | -7 | ATG/TAA |
| *COX3* | 9078 | 9862 | H | 785 | 59 | ATG/TA |
| *tRNA-Gly* | 9863 | 9933 | H | 71 |  |  |
| *ND3* | 9934 | 10282 | H | 349 |  | ATA/T |
| *tRNA-Arg* | 10283 | 10351 | H | 69 |  |  |
| *nad4l* | 10352 | 10648 | H | 297 |  | ATG/TAA |
| *ND4* | 10642 | 12022 | H | 1381 | -7 | ATG/T |
| *tRNA-His* | 12023 | 12092 | H | 70 |  |  |
| *tRNA-Ser* | 12093 | 12159 | H | 67 |  |  |
| *tRNA-Leu* | 12163 | 12234 | H | 72 | 3 |  |
| *ND5* | 12235 | 14100 | H | 1866 |  | ATG/TAA |
| *ND6* | 14071 | 14592 | L | 522 | -30 | ATG/TAG |
| *tRNA-Glu* | 14593 | 14661 | L | 69 |  |  |
| *cytb* | 14667 | 15807 | H | 1141 | 5 | ATG/T |
| *tRNA-Thr* | 15808 | 15879 | H | 72 |  |  |
| *tRNA-Pro* | 15879 | 15948 | L | 70 | -1 |  |
| NCR | 15949 | 17286 | H | 1338 |  |  |

* Intergenic Region: Negative numbers indicate overlapping nucleotides between adjacent genes.

**Table S5.** Base composition of the *Neobythites nanhaiensis* sp. nov. mitochondrial genome.

| **Regions** | ***Neobythites nanhaiensis* sp. nov. (voucher no. ECSFRI 28760)** | | | | | | | |
| --- | --- | --- | --- | --- | --- | --- | --- | --- |
|  | **Size (bp)** | **T%** | **C%** | **A%** | **G%** | **AT(%)** | **AT skew** | **GC skew** |
| Full genome | 17287 | 24.8 | 28.9 | 30.3 | 16.0 | 55.1 | 0.098 | -0.288 |
| PCGs | 11493 | 26.3 | 30.4 | 27.2 | 16.1 | 53.5 | 0.016 | -0.307 |
| rRNAs | 2625 | 20.9 | 24.2 | 34.1 | 20.8 | 55.0 | 0.241 | -0.075 |
| tRNAs | 1548 | 27.3 | 21.1 | 27.7 | 23.8 | 55.0 | 0.007 | 0.060 |
| 1st codon position | 3831 | 21.0 | 26.2 | 27.1 | 25.6 | 48.1 | 0.126 | -0.012 |
| 2nd codon position | 3831 | 39.7 | 28.5 | 18.5 | 13.3 | 58.2 | -0.364 | -0.364 |
| 3rd codon position | 3831 | 18.2 | 36.5 | 35.9 | 9.4 | 54.1 | 0.328 | -0.589 |
| 12S rRNA | 949 | 20.8 | 25.2 | 31.9 | 22.1 | 52.7 | 0.212 | -0.065 |
| 16S rRNA | 1676 | 20.9 | 23.6 | 35.4 | 20.0 | 56.3 | 0.256 | -0.082 |
| *ATP6* | 693 | 23.8 | 34.9 | 27.3 | 14.0 | 51.1 | 0.068 | -0.428 |
| *ATP8* | 177 | 23.2 | 31.6 | 30.5 | 14.7 | 53.7 | 0.137 | -0.366 |
| *COX1* | 1551 | 28.2 | 28.2 | 25.2 | 18.4 | 53.4 | -0.056 | -0.212 |
| *COX2* | 691 | 24.3 | 29.4 | 30.2 | 16.1 | 54.5 | 0.109 | -0.293 |
| *COX3* | 785 | 25.4 | 31.8 | 25.4 | 17.5 | 50.8 | 0.000 | -0.292 |
| *Cytb* | 1141 | 27.1 | 31.6 | 26.6 | 14.6 | 53.7 | -0.008 | -0.367 |
| *ND1* | 975 | 27.0 | 30.9 | 26.3 | 15.9 | 53.3 | -0.013 | -0.320 |
| *ND2* | 1073 | 24.1 | 31.6 | 29.7 | 14.5 | 53.8 | 0.104 | -0.370 |
| *ND3* | 349 | 26.6 | 33.5 | 23.2 | 16.6 | 49.8 | -0.069 | -0.337 |
| *ND4* | 1381 | 25.1 | 31.1 | 29.3 | 14.5 | 54.4 | 0.077 | -0.364 |
| *ND4L* | 297 | 26.9 | 35.0 | 24.2 | 13.8 | 51.1 | -0.053 | -0.434 |
| *ND5* | 1866 | 24.7 | 31.0 | 30.7 | 13.6 | 55.4 | 0.107 | -0.390 |
| *ND6* | 522 | 39.8 | 14.4 | 14.2 | 31.6 | 54.0 | -0.475 | 0.375 |
| NCR | 1338 | 31.2 | 22.9 | 35.9 | 9.9 | 67.1 | 0.071 | -0.395 |

**Table S6.** Codon number and RSCU of *Neobythites nanhaiensis* sp. nov. mitochondrial PCGs.

| **Codon** | **Count** | **RSCU** | **Codon** | **Count** | **RSCU** | **Codon** | **Count** | **RSCU** | **Codon** | **Count** | **RSCU** |
| --- | --- | --- | --- | --- | --- | --- | --- | --- | --- | --- | --- |
| UUU(F) | 86 | 0.76 | UCU(S) | 43 | 1.02 | UAU(Y) | 39 | 0.64 | UGU(C) | 7 | 0.56 |
| UUC(F) | 140 | 1.24 | UCC(S) | 86 | 2.04 | UAC(Y) | 82 | 1.36 | UGC(C) | 18 | 1.44 |
| UUA(L) | 95 | 0.91 | UCA(S) | 57 | 1.35 | UAA(*) | 6 | 3.43 | UGA(W) | 98 | 1.75 |
| UUG(L) | 22 | 0.21 | UCG(S) | 12 | 0.28 | UAG(*) | 1 | 0.57 | UGG(W) | 14 | 0.25 |
| CUU(L) | 82 | 0.79 | CCU(P) | 39 | 0.73 | CAU(H) | 30 | 0.55 | CGU(R) | 9 | 0.46 |
| CUC(L) | 129 | 1.24 | CCC(P) | 97 | 1.80 | CAC(H) | 80 | 1.45 | CGC(R) | 23 | 1.16 |
| CUA(L) | 239 | 2.30 | CCA(P) | 59 | 1.10 | CAA(Q) | 79 | 1.66 | CGA(R) | 32 | 1.62 |
| CUG(L) | 56 | 0.54 | CCG(P) | 20 | 0.37 | CAG(Q) | 16 | 0.34 | CGG(R) | 15 | 0.76 |
| AUU(I) | 130 | 0.98 | ACU(T) | 40 | 0.51 | AAU(N) | 37 | 0.62 | AGU(S) | 11 | 0.26 |
| AUC(I) | 136 | 1.02 | ACC(T) | 135 | 1.73 | AAC(N) | 82 | 1.38 | AGC(S) | 44 | 1.04 |
| AUA(M) | 149 | 1.48 | ACA(T) | 129 | 1.65 | AAA(K) | 63 | 1.48 | AGA(*) | 0 | 0 |
| AUG(M) | 52 | 0.52 | ACG(T) | 9 | 0.12 | AAG(K) | 22 | 0.52 | AGG(*) | 0 | 0 |
| GUU(V) | 44 | 0.85 | GCU(A) | 46 | 0.50 | GAU(D) | 23 | 0.61 | GGU(G) | 30 | 0.5 |
| GUC(V) | 43 | 0.83 | GCC(A) | 160 | 1.75 | GAC(D) | 52 | 1.39 | GGC(G) | 91 | 1.53 |
| GUA(V) | 91 | 1.77 | GCA(A) | 146 | 1.60 | GAA(E) | 76 | 1.57 | GGA(G) | 56 | 0.94 |
| GUG(V) | 28 | 0.54 | GCG(A) | 13 | 0.14 | GAG(E) | 21 | 0.43 | GGG(G) | 61 | 1.03 |

*Stop codon
